# Cataract Surgery and Visual Acuity in Elderly Japanese: Results of Fujiwara-kyo Eye Study

**DOI:** 10.1089/biores.2017.0007

**Published:** 2017-04-01

**Authors:** Kimie Miyata, Tadanobu Yoshikawa, Masashi Mine, Tomo Nishi, Nozomi Okamoto, Tetsuo Ueda, Ryo Kawasaki, Norio Kurumatani, Nahoko Ogata

**Affiliations:** ^1^ Department of Ophthalmology, Nara Medical University, Kashihara, Japan.; ^2^ Department of Epidemiology and Preventive Medicine, Nara Medical University, Kashihara, Japan.; ^3^ Department of Public Health, Graduate School of Medical Science, Yamagata University, Yamagata, Japan.

**Keywords:** cataract, cataract surgery, cohort study, Fujiwara-kyo, visual acuity

## Abstract

The aim of this study was to determine the presence of prior cataract surgery and best-corrected visual acuity (BCVA) in an elderly Japanese cohort. The Fujiwara-kyo Eye Study was a prospective, population-based, cross-sectional epidemiological study. The subjects were ≥68 years who lived in the Nara Prefecture and responded to recruitment notices. All of the subjects underwent comprehensive ophthalmological examinations, and the sociodemographic information and medical history, including prior cataract surgery, were obtained by answers to a questionnaire. The associations between the BCVA, age, sex, and history of cataract surgery were determined. A total of 2,873 subjects whose mean age was 76.3 ± 4.9 (mean ± standard deviation) years were studied. The mean BCVA was −0.020 ± 0.14 logarithm of the minimum angle of resolution units, and it was significantly better in the group with education ≥13 years (*p* < 0.01). Overall, 24.2% of the subjects had undergone cataract surgery, and 41.7% of the subjects ≥80 years had undergone cataract surgery. The incidence of prior cataract surgery increased with increasing age (*p* < 0.001 for trend). The mean BCVA of eyes with cataract surgery was significantly better than that of eyes without cataract surgery in subjects ≥80 years (*p* < 0.01). Visual acuity was generally good in this cohort of elderly Japanese subjects. In this cohort, 24.2% of the subjects had undergone cataract surgery, and the subjects ≥80 years had better BCVA than those without cataract surgery.

## Introduction

The age of individuals in developed countries is rapidly increasing, and Japan has become a superaged society. The average life span of men in Japan was 80.50 years and that of women was 86.83 years in 2014, which was one of the leading life spans in the world as reported by the Ministry of Health, Welfare, and Labour in Japan (www.mhlw.go.jp/toukei/saikin/hw/life/life13/index.html). According to an estimate by the Cabinet Office of Japan (www8.cao.go.jp/kourei/whitepaper/w-2015/gaiyou/27pdf_indexg.html), the proportion of people aged ≥65 years was 26.5% and that of people ≥75 years was 12.5% in 2014. The proportions are estimated to increase to 31.6% and 19.5%, respectively, in 2030.

To maintain a good quality of life (QOL), individuals must retain good physical and mental health. Vision is an important factor that can help maintain a good QOL in elderly subjects.^1^ An earlier study showed that subjects with good visual acuity after cataract surgery had not only a better QOL^2^ but also a longer life expectancy.^3–5^

Although several epidemiological surveys have been conducted to determine the visual function in the general population, there is still a lack of data on the elderly populations, especially those over 70 years.^6–8^ The Fujiwara-kyo Study is a cohort study that was begun in 2007 and was conducted to investigate the functional capacities and the QOL of elderly subjects in a community in Nara, Japan.^9–11^ As a part of the Fujiwara-kyo Study, we began an ophthalmological survey in 2012, named the Fujiwara-kyo Eye Study, to determine the influence of visual acuity on the functional daily activities and on the QOL.

The purpose of this study was to determine the best-corrected visual acuity (BCVA) and factors associated with BCVA in an elderly cohort. We also determined the relationship between visual acuity and the history of cataract surgery because cataract is the most common eye disorder of the elderly.^3,12–15^

## Subjects and Methods

### The Fujiwara-kyo Study

A detailed description of the Fujiwara-kyo Study has been reported.^9–11^ Briefly, this was a cohort study whose purpose was to identify factors related to the maintenance of a healthy life, prevention of physical weakness, and improvement of the functional capacities and the QOL of an elderly population in Japan.^9–11^ The Fujiwara-kyo Study was initially performed in 2007 on 4,427 individuals. The subjects consisted of residents in Nara Prefecture who were ≥65 years and living independently in their own homes. Nara Prefecture is located in the Western part of Japan and is a suburban city. The population of Nara Prefecture is 1.4 million people.

### The Fujiwara-kyo Eye Study

An ophthalmological examination was not conducted in the initial survey in 2007, and an ophthalmological survey (Fujiwara-kyo Eye Study) was conducted for the first time during the second survey between February and November 2012. The second survey of Fujiwara-kyo Eye Study invited 2,873 subjects, including 80 newly recruited volunteers who were ≥65 years. The data presented in this article were collected in 2012. The subjects recruited at the initial survey in 2007 were ≥65 years and were 5 years older in 2012. There were 80 newly recruited subjects in 2012 who were ≥65 years.

The surveys were conducted in accordance with the tenets of the Declaration of Helsinki, and the protocol was approved by the Ethics Review Board of Nara Medical University. A signed informed consent form was obtained from all participants.

### Ophthalmological examinations

#### Self-administered ophthalmological questionnaires

A history of cataract surgery was determined by a self-administered questionnaire. The eyes were divided into those with prior cataract surgery and those without. The subjects who had undergone cataract surgery in at least one eye were placed in the cataract surgery group for the individual analyses. Two hundred four randomly selected participants were examined by slit lamp to verify the accuracy of self-administered questionnaires regarding cataract surgery. For this, we checked the status of the lens or the intraocular lens to confirm an agreement with the self-administered questionnaires. The Kappa coefficient between self-administered questionnaires and diagnosis based on the slit lamp examination was high at 0.95.

#### Visual acuity

The uncorrected and corrected visual acuities of both eyes were measured with a Landolt ring chart at 5 m. The refractive errors were determined by an autorefractometer and keratometer (ARK-700A; Nidek, Gamagori, Japan). The visual acuity was measured according to the standard of the International Organization for Standardization.^16^ The decimal BCVA was converted to the logarithm of the minimum angle of resolution (logMAR) units for statistical analyses. To describe the age-specific BCVA, subjects were divided into five age groups, viz., ≤74 years, 75–79 years, 80–84 years, 85–89 years, and ≥90 years.

Subjects with a BCVA of <20/40 (>0.3 logMAR units) in the better seeing eye, the criterion of vision reduction used in the Blue Mountains Eye Study,^7^ was classified as being visually impaired. Blindness was defined as a BCVA in the better seeing eye of <3/60 based on the World Health Organization standards.

### Statistical analyses

The significance of the differences in age and BCVA between sexes was tested by unpaired *t* tests as they were considered to be normally distributed. The significance of the differences in the number of eyes or subjects with Snellen visual acuity <20/40 in men and women was determined by chi-square tests. The BCVA of the different ages was analyzed by linear regression analysis. The associations between BCVA and the sociodemographics and health characteristics of the subjects were analyzed by unpaired *t* tests. The age-adjusted mean of BCVA was analyzed by analysis of covariance. The percentage of subjects with cataract surgery in the groups as a function of age was analyzed by linear regression analysis. The BCVA of eyes with and without cataract surgery in groups classified by age was analyzed by unpaired *t* tests. The differences of the sociodemographic and health characteristics of individuals with and without cataract surgery were analyzed by chi-square and unpaired *t* tests. Statistical analyses were performed with SPSS (version 22.0; SPSS, Inc., Chicago, IL). A *p* < 0.05 was taken to be significant.

## Results

A total of 2,873 subjects with a mean age of 76.3 ± 4.9 years (range 68–100 years) were studied in the Fujiwara-kyo Eye Study. There were 1,514 men (52.6%) whose mean age was 76.5 ± 4.9 (mean ± standard deviation) years and 1,359 women (47.3%) whose mean age was 76.0 ± 4.8 years. The mean BCVA of the better eye of the 2,873 subjects was −0.020 ± 0.14 logMAR units, of the men was −0.028 ± 0.13 logMAR units, and that for the women was −0.010 ± 0.14 logMAR units. The BCVA was significantly better in men than in women (*p* < 0.01; Table 1). A visual impairment of the better eye was observed in 97 of the 2,873 subjects (3.4%; Table 1), and the incidence was significantly higher in women than in men (*p* < 0.01; Table 1). None of the subjects were classified as being blind according to our definition.

**Table 1.** **Basic Characteristics of 2,873 Subjects**

Characteristics	All, *n* = 2,873	Male, *n* = 1,514	Female, *n* = 1,359	*p*
Age, mean ± SD, years	76.3 ± 4.9	76.5 ± 4.9	76.0 ± 4.8	<0.05
BCVA of better seeing eye, mean ± SD, LogMAR units	−0.020 ± 0.14	−0.028 ± 0.13	−0.010 ± 0.14	<0.01
BCVA ≥0.3 LogMAR units, number of subjects (%)	97 (3.4)	36 (2.4)	61 (4.5)	<0.01

BCVA, best-corrected visual acuity; logMAR, logarithm of the minimum angle of resolution; SD, standard deviation.

### Visual acuity by age groups

**FIG. 1.**
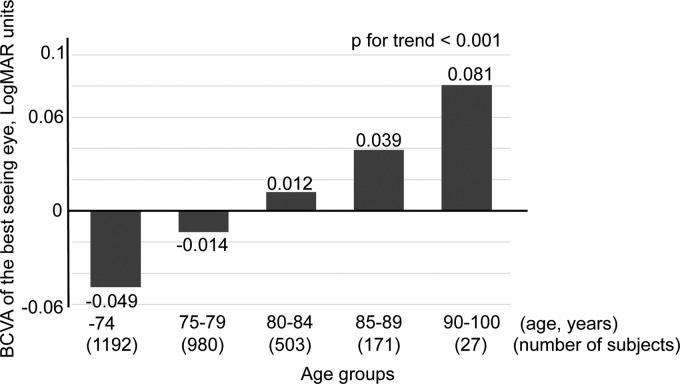
The BCVA of the better eye for different ages. The BCVA (mean ± standard deviation) was −0.049 ± 0.13 logMAR units in those aged ≤74 years (1,192 subjects), −0.014 ± 0.13 logMAR units in those 75–79 years (980 subjects), 0.012 ± 0.13 logMAR units in those 80–84 years (503 subjects), 0.039 ± 0.16 logMAR units in those in 80–84 years (171 subjects), and 0.081 ± 0.15 logMAR units in those ≥90 years (27 subjects). The BCVA was significantly worse in the older subjects (*p* < 0.01 for trend, analysis of variance). BCVA, best-corrected visual acuity; logMAR, logarithm of the minimum angle of resolution.

The subjects were divided into five groups by age. The mean BCVA was −0.049 ± 0.13 logMAR units in those ≤74 years, −0.014 ± 0.13 logMAR units in the 75–79-year group, 0.012 ± 0.13 logMAR units in the 80–84-year group, 0.039 ± 0.16 logMAR units in the 80–84-year group, and 0.081 ± 0.15 logMAR units in the ≥90-year group. Linear regression analyses showed that BCVA was significantly worse in the older subjects (*p* < 0.01 for trend; Fig. 1). The BCVA was 0.021 ± 0.14 logMAR units in those aged ≥80 years (701 subjects).

### Population of subjects with prior cataract surgery by age

**FIG. 2.**
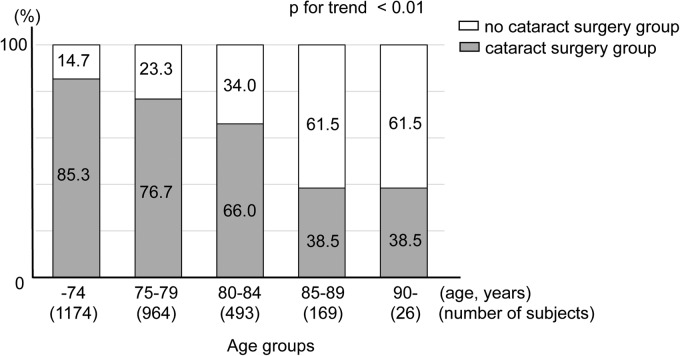
Percentage of subjects with prior cataract surgery as a function of age. A total of 685 subjects (24.2%) had undergone cataract surgery. The percentage of subjects who had undergone cataract surgery was 14.7% in those aged ≤74 years (1,174 subjects), 23.3% in those 75–79 years (964 subjects), 34.3% in those 80–84 years (493 subjects), 61.5% in those 80–84 years (169 subjects), and 61.5% in those ≥90 years (26 subjects). The percentages of subjects who had had cataract surgery increased significantly with increasing age (*p* < 0.01 for trend, linear regression analysis).

The history of cataract surgery was not obtained from 47 subjects because of a failure to answer the questions or failure to be recorded. There were 685 subjects (24.2%) in the prior cataract surgery group and 2,141 subjects (75.8%) in the no cataract surgery group (Table 4). The percentage of subjects who had undergone cataract surgery was 14.7% in the ≤74-year group, 23.3% in the 75–79-year group, 34.0% in the 80–84-year group, 61.5% in the 85–90-year group, and 61.5% in the ≥90-year group. The percentage of subjects who had undergone cataract surgery increased with age (*p* < 0.01 for trend; Fig. 2), and 41.7% of subjects aged ≥80 years had had cataract surgery.

### Correlations between BCVA and the socio-demographics and health characteristics

The mean BCVA of the better-seeing eye was significantly better in the group with ≥13 years of education than that in the group with ≤12 years of education (BCVA, −0.039 ± 0.14 vs. −0.015 ± 0.13 logMAR units, *p* < 0.01; Table 2). The differences were still significant when they were adjusted by age (*p* < 0.01; Table 2).

**Table 2.** **Associations Between Best-Corrected Visual Acuity and Sociodemographic Data and Health Characteristics of 2,829 Subjects**

	BCVA, mean ± SD, LogMAR units			Age adjusted mean, LogMAR units		
	Present	Absent	*p*	Present	Absent	*p*
BMI ≥25 kg/m^2^ (% of subjects)	−0.023 ± 0.13 (19.4)	−0.020 ± 0.14 (80.6)	0.6	−0.020	−0.020	0.9
Education ≥13 years (% of subjects)	−0.039 ± 0.14 (22.9)	−0.015 ± 0.13 (77.1)	<0.01	−0.038	−0.015	<0.01
Current smoking (% of subjects)	−0.019 ± 0.12 (5.9)	−0.020 ± 0.14 (94.1)	0.9	−0.016	−0.020	0.7
History of disease stroke (% of subjects)	0.0002 ± 0.14 (6.1)	−0.021 ± 0.14 (93.9)	0.04	−0.008	−0.021	0.2
Myocardial infarction (% of subjects)	−0.013 ± 0.11 (2.8)	−0.020 ± 0.14 (97.2)	0.7	−0.020	−0.022	0.9
Angina (% of subjects)	−0.023 ± 0.12 (8.2)	−0.020 ± 0.14 (91.8)	0.7	−0.028	−0.019	0.4
Hypertension (% of subjects)	−0.020 ± 0.13 (51.8)	−0.021 ± 0.14 (48.2)	0.9	−0.022	−0.018	0.4
Diabetes mellitus (% of subjects)	−0.011 ± 0.13 (15.0)	−0.022 ± 0.14 (85.0)	0.2	−0.011	−0.022	0.14

BMI, body–mass index.

### BCVA of eyes with and without cataract surgery in age groups

The differences in BCVA between eyes with prior cataract surgery and without cataract surgery were not significant in groups under 80 years. However, the eyes of subjects ≥80 years who had undergone cataract surgery had better BCVA compared with eyes without cataract surgery (*p* < 0.01; Table 3).

**Table 3.** **Best-Corrected Visual Acuity of Eyes With and Without Cataract Surgery in Groups Classified by Age**

	BCVA, mean ± SD, LogMAR units		
Age group years (no. of eyes)	Eyes with cataract surgery (no. of eyes)	Eyes with no cataract surgery (no. of eyes)	*p*
≤74 (2,346)	0.031 ± 0.33 (287)	0.007 ± 0.23 (2,059)	0.2
75–79 (1,925)	0.053 ± 0.32 (385)	0.060 ± 0.25 (1,540)	0.6
80–84 (986)	0.033 ± 0.22 (293)	0.107 ± 0.25 (693)	<0.01
85–89 (338)	0.068 ± 0.24 (190)	0.182 ± 0.34 (148)	<0.01
90≥ (52)	0.095 ± 0.20 (29)	0.279 ± 0.31 (23)	0.01

### Sociodemographic characteristics and cataract surgery

The incidence of prior cataract surgery in men was significantly lower than that of women (*p* < 0.01; Table 4). The percentage and odds ratio (OR) of subjects with prior stroke or had hypertension and diabetes mellitus were higher in subjects who had prior cataract surgery (*p* < 0.02, OR 1.47, *p* < 0.04, OR 1.20, and *p* < 0.01, OR 1.40, respectively; Table 4). The prevalence of prior cataract surgery was not significantly associated with the duration of education.

**Table 4.** **Sociodemographic Data and Health Characteristics of Subjects with Cataract Surgery and with No Cataract Surgery**

	No. of subjects, *n* = 2,826	Cataract surgery no. of subjects (%), *n* = 685	No cataract surgery no. of subjects (%), *n* = 2,141	*p*	OR	95% CI
Age, mean ± SD, years	2,826	78.8 ± 5.4	75.5 ± 4.4	<0.01	2.63	2.17–3.19
Gender, men	1,487	332 (48.5)	1,155 (53.9)	0.01	1.22	1.04–1.47
BMI ≥25 kg/m^2^	547	126 (18.4)	421 (19.7)	0.5	0.92	0.74–1.15
Education ≥13 years	692	189 (27.6)	503 (23.5)	0.2	1.00	1.00–1.00
Current smoking, present	164	34 (5.0)	130 (6.1)	0.3	0.81	0.55–1.19
History of disease						
Stroke, present	175	55 (8.0)	120 (5.6)	0.02	1.47	1.06–2.05
Myocardial infarction, present	80	26 (3.8)	54 (2.5)	0.09	0.65	0.41–1.65
Angina, present	233	58 (8.5)	175 (8.2)	0.8	0.96	0.70–1.31
Hypertension, present	1466	379 (55.3)	1,087 (50.7)	0.04	1.20	1.01–1.43
Diabetes mellitus, present	424	126 (18.4)	298 (13.9)	<0.01	1.40	1.11–1.76

CI, confidence interval; OR, odds ratio.

## Discussion

The mean BCVA of −0.020 ± 0.14 logMAR units for all of the participants indicated that the visual acuity was generally good in this elderly cohort. There were only 3.4% of participants with a visual impairment defined as having a fully corrected Snellen visual acuity of <20/40 (>0.3 logMAR units). The BCVA was significantly worse in the older subjects. The prevalence of visual impairments (visual acuity <20/40) in the better eye was 12.3% for those ≥55 years in the Blue Mountains Eye Study^17^ and it was 27.8% in the ≥80-year group in the Los Angeles Latino Eye Study.^18^ Compared with these figures, it is noteworthy that a mean BCVA of 0.021 logMAR units in subjects ≥80 years is relatively good as has been reported in an earlier study on Japanese individuals.^19^

It is interesting that the BCVA adjusted by age was significantly better in subjects with education ≥13 years than with ≤12 years of education. A recent study reported that the prevalence of any visual impairment (Snellen visual acuity <20/40) was two times higher in the lower socioeconomic group.^20^ Our data are in agreement with these results if we assume that a shorter education time reflects a lower socioeconomic status.

The sociodemographic and health characteristics of the subjects who had undergone cataract surgery have been reported.^5,21,22^ In this study, the subjects who had prior cataract surgery were significantly older, were more likely to be women, and had a higher incidence of stroke, hypertension, and diabetes than in subjects without prior cataract surgery. An earlier study reported that the subjects in the longer duration education group had a higher chance of having had cataract surgery^22^; however, we did not find a significant association between the duration of education and a history of cataract surgery.

In our cohort, 24.2% had undergone cataract surgery, and almost twice as many subjects (41.7%) aged ≥80 years had undergone cataract surgery. The Singapore Epidemiology of Eye Disease Study reported that the prevalence of cataract surgery in people aged ≥80 years was 63.9% in the Chinese population, 38.89% in the Malaysian population, and 68.25% in the East Indian population.^21^ These observations are higher than that of the Blue Mountains Eye Study of 23% in the ≥80-year group.^23^ These differences may be due to the sociodemographic circumstances and the systems of health insurance.

Cataract surgery is performed more often in some countries and increasing trends of surgeries are observed in two populations.^23,24^ In the Blue Mountains Eye Study, there was a 32% increase in cataract surgery over a 6-year period from 1992–1994 to 1997–2000 with age-standardized prevalence of 6.0–7.7%. It was more marked among people ≥80 years old from 23.0% to 31.7%.^23^ It has been estimated that the number of subjects with cataract surgery has increased in the last decade reflecting continued aging of the population.^24^ In the United States, the incidence of lens extraction has also increased over the past 20 years in individuals older than 65 years.^25^ Japan has become a superaged society and our findings may reflect this situation, showing that cataract surgery is increasing even in those aged ≥80 years.

As expected, eyes with cataracts have greater improvement in visual acuity after cataract surgery.^2,26^ In our study, there was no significant difference of the mean BCVA between eyes with cataract surgery and in eyes without cataract surgery in relatively younger subjects of <79 years. However, the mean BCVA of eyes with prior cataract surgery was significantly better compared with eyes with no cataract surgery in subjects ≥80 years (*p* < 0.01). These results may indicate that relatively younger subjects are concerned about failing vision even if only slightly, and they undergo cataract surgery at an earlier age. Alternatively, subjects ≥80 years may not wish to undergo cataract surgery even if they have failing vision.

It has been reported that the life expectancy was longer in patients whose visual acuity was better after cataract surgery.^3–5,27^ We found in an earlier cross-sectional study that subjects with cataract surgery had significantly lower age-adjusted ORs for cognitive impairment than subjects without cataract surery.^28^ In addition, we recently reported that subjects with visual impairment had twofold higher OR for the prevalence of cognitive impairment (Snellen visual acuity <20/29) than subjects without visual impairment.^29^ Thus, we believe that improvements of visual acuity after cataract surgery may have beneficial effects on both the QOL and longevity.

There are limitations in this study. Subjects were not randomly selected from the geographical area as it was based on participants who volunteered. Thus, there is a chance of introducing selection bias of having relatively healthier and more independent persons. In addition, the causes of visual impairment were not determined in detail. Because the primary aim of the Fujiwara-kyo Study was to survey the functional capabilities and QOL of independent elderly residents in a community, ophthalmological examinations were limited to determining only the visual functions in these elderly subjects.

In conclusion, we found that 1 in 4 (24.2%) of the subjects aged ≥68 years had undergone cataract surgery, and the subjects aged ≥80 years had better BCVA than those without cataract surgery. This study is the first to report the prevalence of cataract surgery in elderly subjects in a relatively large sample size in Japan. Future follow-up examinations will reveal the associations of visual functions, especially whether visual acuity contributes to maintain good QOL and healthy life expectancy.

## Abbreviations Used

BCVA: best-corrected visual acuity
BMI: body–mass index
CI: confidence interval
logMAR: logarithm of the minimum angle of resolution
OR: odds ratio
QOL: quality of life
SD: standard deviation

## References

[1] Stuck AE, Walthert JM, Nikolaus T, et al. Risk factors for functional status decline in community-living elderly people: a systematic literature review. Soc Sci Med. 1999;48:445–469 10075171 10.1016/s0277-9536(98)00370-0

[2] Chandrasekaran S, Wang JJ, Rochtchina E, et al. Change in health-related quality of life after cataract surgery in a population-based sample. Eye (Lond). 2008;22:479–484 17479118 10.1038/sj.eye.6702854

[3] Fong CS, Mitchell P, Rochtchina E, et al. Correction of visual impairment by cataract surgery and improved survival in older persons: the Blue Mountains Eye Study cohort. Ophthalmology. 2013;120:1720–1727 23664468 10.1016/j.ophtha.2013.02.009

[4] Fong CS, Mitchell P, Rochtchina E, et al. Visual impairment corrected via cataract surgery and 5-year survival in a prospective cohort. Am J Ophthalmol. 2014;157:163–170 24161249 10.1016/j.ajo.2013.08.018

[5] Tseng VL, Yu F, Lum F, et al. Cataract surgery and mortality in the United States Medicare Population. Ophthalmology. 2016;123:1019–1026 26854033 10.1016/j.ophtha.2015.12.033

[6] Klein R, Klein BE, Linton KL, et al. The Beaver Dam Eye Study: visual acuity. Ophthalmology. 1991;98:1310–1315 1923372 10.1016/s0161-6420(91)32137-7

[7] Attebo K, Mitchell P, Smith W Visual acuity and the causes of visual loss in Australia. The Blue Mountains Eye Study. Ophthalmology. 1996;103:357–364 8600410 10.1016/s0161-6420(96)30684-2

[8] Azen SP, Varma R, Preston-Martin S, et al. Binocular visual acuity summation and inhibition in an ocular epidemiological study: the Los Angeles Latino Eye Study. Invest Ophthalmol Vis Sci. 2002;43:1742–1748 12036974

[9] Iki M, Fujita Y, Tamaki J, et al. Design and baseline characteristics of a prospective cohort study for determinants of osteoporotic fracture in community-dwelling elderly Japanese men: the Fujiwara-kyo osteoporosis risk in men (FORMEN) study. BMC Musculoskelet Disord. 2009;10:165 20030855 10.1186/1471-2474-10-165 PMC2811107

[10] Nezu S, Okamoto N, Morikawa M, et al. Health-related quality of life (HRQOL) decreases independently of chronic conditions and geriatric syndromes in older adults with diabetes: the Fujiwara-kyo Study. J Epidemiol. 2014;24:259–266 24814506 10.2188/jea.JE20130131 PMC4074629

[11] Okamoto N, Morikawa M, Tomioka K, et al. Association between tooth loss and the development of mild memory impairment in the elderly: the Fujiwara-kyo Study. J Alzheimers. 2015;44:777–786 10.3233/JAD-141665 25362033

[12] Wang JJ, Foran S, Mitchell P Age-specific prevalence and causes of bilateral and unilateral visual impairment in older Australians: the Blue Mountains Eye Study. Clin Experiment Ophthalmol. 2000;28:268–273 11021555 10.1046/j.1442-9071.2000.00315.x

[13] Foran S, Wang JJ, Mitchell P Causes of visual impairment in two older population cross-sections: the Blue Mountains Eye Study. Ophthalmic Epidemiol. 2003;10:215–225 14628964 10.1076/opep.10.4.215.15906

[14] Tan AG, Wang JJ, Rochtchina E, et al. Comparison of age-specific cataract prevalence in two population-based surveys 6 years apart. BMC Ophthalmol. 2006;6:17 16623958 10.1186/1471-2415-6-17 PMC1524813

[15] Kanthan GL, Wang JJ, Rochtchina E, et al. Ten-year incidence of age-related cataract and cataract surgery in an older Australian population. The Blue Mountains Eye Study. Ophthalmolog. 2008;115:808–814 10.1016/j.ophtha.2007.07.008 17900695

[16] International Organization for Standardization. Ophthalmic Optics—Visual Acuity Testing—Standard Optotype and Its Presentation. ISO. 2009;8596:1–5

[17] Hong T, Mitchell P, Burlutsky G, et al. Visual impairment and the incidence of falls and fractures among older people: longitudinal findings from the Blue Mountains Eye Study. Invest Ophthalmol Vis Sci. 2014;55:7589–7593 25370514 10.1167/iovs.14-14262

[18] Varma R, Ying-Lai M, Klein R, et al. Prevalence and risk indicators of visual impairment and blindness in Latinos: the Los Angeles Latino Eye Study. Ophthalmology. 2004;111:1132–1140 15177963 10.1016/j.ophtha.2004.02.002

[19] Iwano M, Nomura H, Ando F, et al. Visual acuity in a community-dwelling Japanese population and factors associated with visual impairment. Jpn J Ophthalmol. 2004;48:37–43 14767649 10.1007/s10384-003-0013-3

[20] Wah W, Earnest A, Sabanayagam C, et al. Composite measures of individual and area-level socio-economic status are associated with visual impairment in Singapore. PLoS One. 2015;10:e0142302 26555141 10.1371/journal.pone.0142302 PMC4640712

[21] Lavanya R, Wong TY, Aung T, et al. SiMES team. Prevalence of cataract surgery and post-surgical visual outcomes in an urban Asian population: the Singapore Malay Eye Study. Br J Ophthalmol. 2009;93:299–304 18927226 10.1136/bjo.2008.148650

[22] Chua J, Koh JY, Tan AG, et al. Ancestry, socioeconomic status, and age-related cataract in Asians: The Singapore Epidemiology of Eye Diseases Study. Ophthalmology. 2015;122:2169–2178 26256834 10.1016/j.ophtha.2015.06.052

[23] Tan AG, Wang JJ, Rochtchina E, et al. Increase in cataract surgery prevalence from 1992–1994 to 1997–2000: analysis of two population cross-sections. Clin Experiment Ophthalmol. 2004;32:284–288 15180841 10.1111/j.1442-9071.2004.00817.x

[24] Rochtchina E, Mukesh BN, Wang JJ, et al. Projected prevalence of age-related cataract and cataract surgery in Australia for the years 2001 and 2021: pooled data from two population-based surveys. Clin Experiment Ophthalmol. 2003;31:233–236 12786774 10.1046/j.1442-9071.2003.00635.x

[25] Klein BE, Howard KP, Lee KE, et al. Changing incidence of lens extraction over 20 years: the Beaver Dam eye study. Ophthalmology. 2014;121:5–9 23932514 10.1016/j.ophtha.2013.06.006 PMC3830736

[26] Desapriya E, Subzwari S, Scime-Beltrano G, et al. Vision improvement and reduction in falls after expedited cataract surgery Systematic review and metaanalysis. J Cataract Refract Surg. 2010;36:13–19 20117700 10.1016/j.jcrs.2009.07.032

[27] Blundell MS, Hunt LP, Mayer EJ, et al. Reduced mortality compared with national averages following phacoemulsification cataract surgery: a retrospective observational study. Br J Ophthalmol. 2009;93:290–295 18838408 10.1136/bjo.2008.141473

[28] Miyata K, Obayashi K, Saeki K, et al. Higher cognitive function in elderly individuals with previous cataract Surgery: cross-sectional association independent of visual acuity in the HEIJO-KYO Cohort. Rejuvenation Res. 2016;19:239–243 26414122 10.1089/rej.2015.1718

[29] Mine M, Miyata K, Morikawa M, et al. Association of visual acuity and cognitive impairment in older individuals: Fujiwara-kyo Eye Study. Biores Open Access. 2016;5:228–234 27610269 10.1089/biores.2016.0023 PMC5003003

